# Effect of immersion in different media on the color stability, surface roughness, and microhardness of flowable nanohybrid resin composites

**DOI:** 10.1186/s12903-025-07555-1

**Published:** 2026-01-12

**Authors:** Hend Yousri Abd-Elfattah, Mohamed Elshirbeny Elawsya, Abeer ElSayed ElEmbaby

**Affiliations:** 1 Department of Conservative Dentistry, Faculty of Dentistry, Mansoura University, Mansoura, Egypt; 2 Faculty of Dentistry, Mansoura National University, Gamasa, Egypt; 3 Department of Conservative Dentistry, Faculty of Dentistry, Mansoura University, Algomhoria Street, P.O. Box 35516, Mansoura, Aldakhlia, Egypt

**Keywords:** Color stability, Flowable resin composites, Immersion media, Surface microhardness, Surface roughness

## Abstract

**Background:**

To evaluate color stability, surface roughness, and microhardness of flowable nanohybrid resin composites following immersion in different media.

**Methods:**

Polofil NHT Flow (VOCO GmbH), G-aenial Universal Injectable (GC DENTAL PRODUCTS CORP), and Tetric N-Ceram (Ivoclar Vivadent) were used. From each tested resin composite, 50 disc-shaped specimens (10-mm diameter × 2-mm thickness) were performed. Each group was categorized into five subgroups according to immersion media (*n* = 10): (1) control (without immersion), (2) artificial saliva, (3) coffee, (4) cola, and (5) energy drink. Color stability, surface roughness, and microhardness were evaluated. Data were analyzed through two-way ANOVA, Tukey’s post-hoc, and Pearson correlation coefficient tests (*p* < 0.05).

**Results:**

For color change, no statistically significant differences were observed among the resin composites regarding artificial saliva (*p* = 0.78), coffee (*p* = 0.93), and cola (*p* = 0.09), while there was a significant difference between them regarding energy drink (*p* < 0.05). The highest surface roughness mean value was recorded with Polofil NHT Flow, which showed an increase in surface roughness after immersion in energy drink (0.32 ± 0.06 μm) compared to the control subgroup (0.22 ± 0.08 μm). For all resin composites, there were significant reductions in microhardness with all immersion media in comparison to the control subgroups (*p* < 0.05).

**Conclusions:**

For color stability, energy drink and coffee had the most negative effect on all tested resin composites. Energy drink caused the highest discoloration with G-aenial Universal Injectable and the highest roughness with Polofil NHT Flow. All tested resin composites showed a decrease in surface microhardness following immersion in all tested immersion media.

## Introduction

Resin-based composites (RBCs) are commonly used for dental restorations due to their favorable esthetic qualities, strong bonding to the tooth substrate, and little necessity of tooth preparation [1, 2]. Since the pursuit of an optimal and pleasing restorative material is nearly as old as dentistry itself, advances in nanohybrid resin composites incorporating fillers that enhance polishability, color stability, esthetics, and mechanical performance have played a key role in meeting these demands [3, 4].

The qualities of their inorganic fillers, especially their size, have an impact on the characteristics of RBCs and can affect the restoration’s longevity and esthetic result. The enhancement of nanoparticle fillers that are integrated into the resin matrix to reduce the stress caused by polymerization shrinkage and reinforce the physio-mechanical characteristics and esthetic appeal of these resin composite materials has been made possible by nanotechnology [5–7].

Similar to conventional types of direct resin composites, the mechanical characteristics are greatly impacted by the amount, type, size, and silanization of the inorganic filler. The type of organic matrix, which determines the mechanical qualities of the organic component, and the inorganic filler, which is defined by its size, shape, and type, are the two primary variables that affect the mechanical characteristics of flowable resin composites. Therefore, new highly filled flowable nanohybrid resin composites that combine high mechanical qualities and flowability have been introduced, and now are widely used for anterior and posterior restorations [8].

The color change of restorative material is a general drawback of resin composites. One of the many possible causes is matrix degradation brought on by hygroscopic water absorption, which reveals the fillers; this is called intrinsic discoloration [9, 10]. Furthermore, the adsorption of the coloring substances found in certain foods and beverages can also result in extrinsic discoloration [11]. Because esthetic restorations are often vulnerable to both intrinsic and external stains, color stability is a main characteristic to take into account. According to reports, colorant-containing foods and beverages can stain the restorative materials, reducing their durability and long-term effectiveness [12–14].

When assessing a dental restoration, surface roughness is a crucial characteristic. This is because a rough surface encourages food particles and dental plaque to accumulate on the restoration and the tooth structure [15, 16]. As a result, secondary caries is initiated, and gingival inflammation occurs. Furthermore, surface roughness results in surface deterioration, discoloration, and a reduction in restoration gloss. Conversely, dental materials’ smooth surfaces could be the cause of their ease of cleaning and less bacterial growth. Because surface roughness degrades the material’s esthetic and biomechanical qualities and increases susceptibility to aging, it has a reputation for being associated with the extended duration of clinical effectiveness of dental restorations [17].

Considering the physical and mechanical characteristics of resin composites, surface microhardness is a crucial element. Surface microhardness is correlated with compressive strength, the degree of conversion, and the resistance to intraoral softening [18–21]. A decrease in surface microhardness raises the risk of dental materials wearing down and becoming fatigued, which can cause the failure of restorations. The material’s composition, the duration of curing, and the environment all affect surface microhardness to some degree [22–25].

In daily life, a wide variety of meals and beverages are consumed in varying amounts, temperatures, colors, and compositions, all of which have an impact on the intraoral structures. For instance, two of the most popular hot and soft drinks, which are frequently consumed, are coffee and cola [26]. Furthermore, because citric acid is found in so many foods and drinks, it is frequently consumed in daily life. Among these are energy drinks, which are becoming more and more popular, particularly among adults between the ages of 18 and 35 [27, 28].

Prolonged usage of these drinks can cause teeth erosion and damage to dental restorations. The demineralization of hard tooth structures and the modification of dental material qualities might result from prolonged exposure to acidic substances that surpass the body’s defenses [29, 30]. Because of superficial degradation or staining solution penetration and sorption in the uppermost layer of resin composite, the external and sublayer structures of resin composite restorations in the oral cavity may become discolored, surface roughness also increases and results in decreased resistance. This can also result in decreasing the microhardness of the resin composite [31, 32].

Although many studies evaluated the effect of different immersion media on the properties of resin composites restorative materials [10–15, 26–29], there is a lack of studies on the effect of different immersion media (particularly energy drinks) on the color stability and surface characteristics of highly filled flowable nanohybrid resin composites. This investigation aimed to resolve this gap through evaluating the influence of various immersion media on the stability of color and surface characteristics of highly filled flowable nanohybrid resin composites. Gaining insight into these effects is essential for anticipating the material’s durability and esthetic performance in the oral environment, ultimately assisting clinicians in material selection and providing dietary recommendations to patients.

The goal of this in vitro study was to evaluate and compare the color stability, surface roughness, and surface microhardness of highly filled flowable nanohybrid resin composites following their immersion in different media. The first null hypothesis proposed that the type of resin composite would not affect color stability, surface roughness, and surface microhardness. The second null hypothesis evaluated was that exposure to different immersion media would not alter the color stability, surface roughness, or surface microhardness of the tested resin composites.

## Materials and methods

### Materials

In the present study, the following resin composite materials were utilized: two commercially available flowable nanohybrid resin composites, Polofil NHT Flow (VOCO GmbH, Cuxhaven, Germany), and G-aenial Universal Injectable (GC DENTAL PRODUCTS CORP, KASUGAI, AICHI, JAPAN), and another conventional nanohybrid resin composite, Tetric N-Ceram (Ivoclar, Vivadent, AG, Schaan/Liechtenstein) as a control. Four different types of immersion media were used: artificial saliva, coffee, cola, and energy drink. Materials and immersion media utilized in this study are presented in Tables 1 and 2, respectively.

**Table 1:** Resin composite materials used in this study

Resin Composite	Manufacturer and Lot No.	Classification	Composition	Shade	Filler Load
Polofil NHT Flow	VOCO GmbH, Anton-Flettner-Straße 1–3, Cuxhaven, Germany. 2,247,542	Flowable light curing nanohybrid resin composite.	Resin matrix: Bis-GMA, TEGDMA, HEDMA, UDMA. Polofil NHT Flow combines nano-scaled particles with conventional sized glass ceramic fillers of a select size range. The result: an extremely high filler content of more than 76%w/w, with an extremely low resin content, compared to other flowable resin composites.	A2	76% w/w
G-aenial Universal Injectable	GC DENTAL PRODUCTS CORP. 2–285 TORIIMATSU-CHO, KASUGAI, AICHI, JAPAN. 2,212,221	Flowable light curing nanohybrid resin composite.	Resin Matrix: UDMA, Bis-MEPP, TEGDMA, Nano- pigment, photoinitiator, A high load of ultra- fine barium particles and GC’s Full -coverage silane coating (FSC) technology with filler 69%w/w.	A2	69% w/w
Tetric N-Ceram	Ivoclar Vivadent AG, Schaan/Liechtenstein. Z056PD	Conventional light curing nanohybrid resin composite.	The resin matrix: Bis-GMA, Bis-EMA and UDMA monomer, Dimethacrylates (19–20%w/w). The fillers: contain barium glass, ytterbium, trifluoride, mixed oxide and copolymer (80–81% w/w).	A2	80–81% w/w

**Table 2:** Immersion media used in this study

Immersion medium	Manufacturer	Ingredient	pH
Artificial saliva	Faculty of Pharmacy, Mansoura University.	1) Ascorbic acid: 0.002 g 2) Glucose: 0.030 g 3) NaCl: 0.580 g 4) CaCl_2_: 0.170 g 5) NH_4_Cl: 0.160 g 6) KCl: 1.270 g 7) NaSCN: 0.160 g 8) KH_2_PO_4_: 0.330 g 9) Urea: 0.200 g 10) Na_2_HPO_4_: 0.340 g 11) Mucin: 2.700 g Distilled water was added to produce one liter of artificial saliva.	7.1
Coffee	Nescafé Classic, Nestlé Egypt.	Water, sugar, milk powder, soluble coffee (2.5%), acidity regulator (E339, E500), flavoring (contains sweetener (E960), emulsifier (E473).	5.1
Cola	Coca-Cola Egypt Company.	Carbonated water, sugar, natural caramel color, acidity regulator (phosphoric acid), kola concentrate, caffeine.	2.8
Red Bull (Energy drink)	Red Bull GmbH, Austria.	Carbonated water, sucrose, glucose, acidity regulator (sodium citrates, magnesium carbonate), caramel color, acidifier citric acid, tourine (0.4%) caffeine (0.03%), artificial dye pigments, vitamin supplements (riboflavin).	3.1

### Methods

#### Study design and sample size calculation

Sample size determination was carried out using data from a prior study [14]. With the G*Power software (version 3.1.9.7), and an effect size of 0.4, a two-tailed test, a significance level of α = 0.01, and a statistical power of 95%, the sample size was calculated to be nine specimens per subgroup. So, ten specimens were included in each subgroup (*n* = 10).

**Fig. 1:**
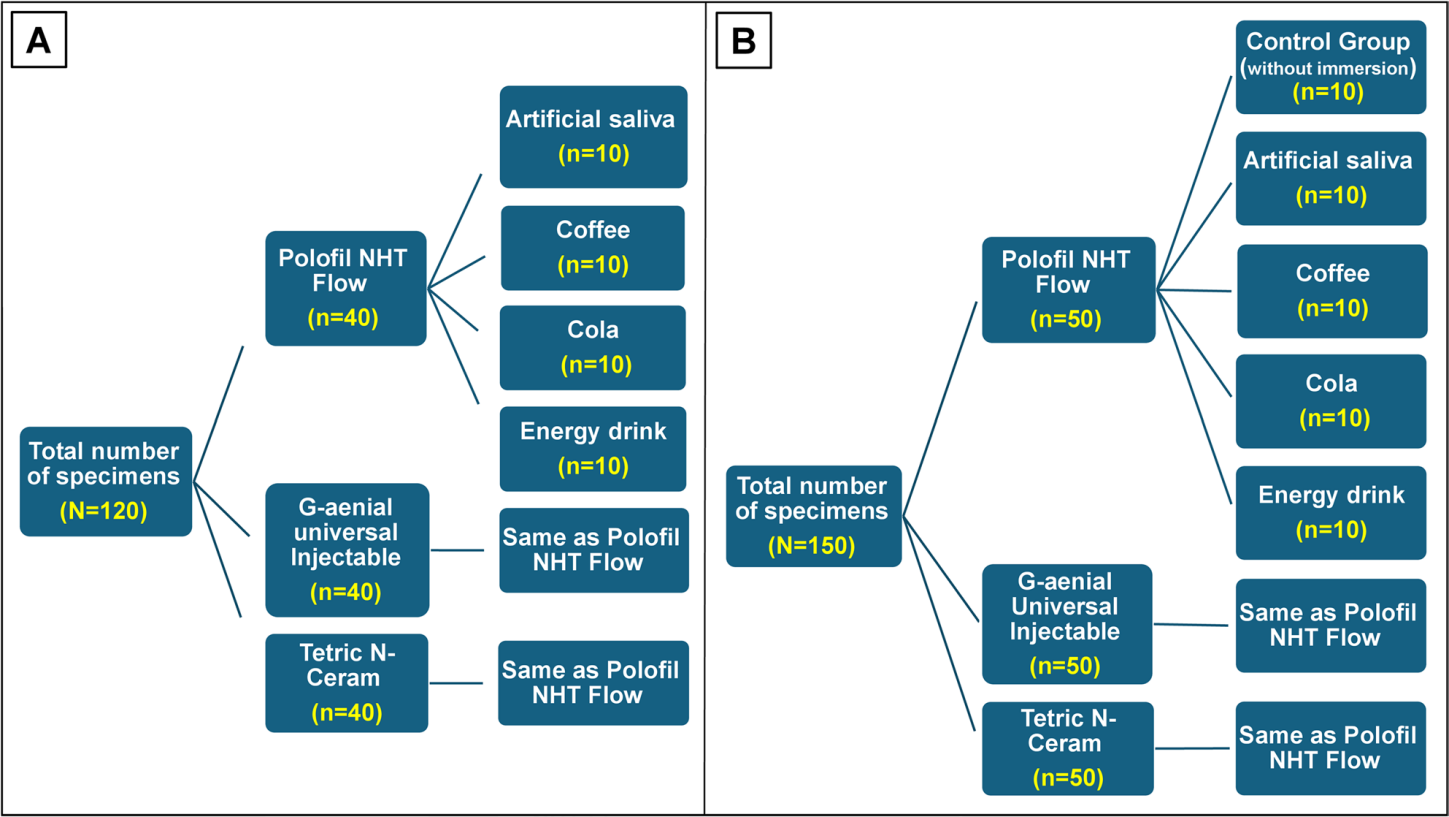
Diagrams showing the study design, **A** for color stability test, **B** for surface roughness and microhardness tests

From each resin composite group, 50 specimens were performed. Five subgroups were divided from each group according to the immersion media (*n* = 10): (1) control (without immersion), (2) artificial saliva, (3) coffee, (4) cola, and (5) energy drink. For the color stability test, all specimens of the immersion subgroups were tested before and after immersion. For surface roughness and microhardness tests, all specimens of the immersion subgroups were tested after immersion in addition to the control subgroups (without immersion). The study design is presented in Fig. 1.

#### Specimen preparation

A standardized split Teflon mold, measuring 10 mm in diameter × 2 mm thickness was used to perform the resin composite specimens. Clear celluloid strips and glass slides were applied to both the upper and lower aspects of the hole in the mold after it was filled with resin composite. In order to accomplish uniform specimen thickness, a constant load of 500 g metal weight was maintained for 30 s on the glass slide positioned above the mold. Each specimen underwent curing from the top surface for 20 s with a light-emitting diode (LED) curing unit (SmartLite focus, Dentsply, USA) with 1,200 mW/cm^2^ irradiance. The light-curing unit output was routinely verified using a radiometer (Bluephase Meter II, Ivoclar Vivadent, Liechtenstein). After curing, to differentiate between the top and bottom surfaces, they were marked from the side of each specimen, then they were polished with a polishing kit (Soflex,3 M ESPE) from the top surface, where color stability, surface roughness, and surface microhardness tests were measured. Specimens were polished in a descending order of grit size using the abrasive discs. Each disc was applied for 20 s with a low-speed handpiece set at 5000 rpm. After every polishing stage, the specimens were thoroughly rinsed under running water for 10 s to eliminate any remaining particles. A fresh disc was used for each specimen to maintain uniformity and prevent contamination. Upon completion of the polishing process, all specimens were stored in distilled water at 37 ± 1 °C for 24 h, then the initial (baseline) color measurements were conducted [33], also surface roughness and microhardness of the control subgroups were measured. All specimens were placed in tightly sealed vials and stored in a dark container in an incubator at 37 ± 1 °C.

### Color stability evaluation

#### Preparation of staining solutions

Following the baseline color measurements, the specimens were exposed to the subsequent staining protocol. The immersion media used in this study and their composition are presented in Table 2.

##### Artificial saliva

One Liter of artificial saliva was prepared in the chemistry laboratory at the Faculty of Pharmacy, Mansoura University, according to a previous study [34].

##### Coffee

One thousand milliliters of boiling water were mixed with twenty grams of coffee (Nescafé Classic, Nestlé Egypt). The solution was mixed every 5 min for 10 s until it reached room temperature, and then the solution was filtered through filter paper [35].

##### Cola

One Liter of cola (Coca-Cola, Egypt).

##### Energy drink

One Liter of Red Bull (Red Bull GmbH, Austria).

Specimens were maintained in an incubator at 37 ± 1 °C after being immersed in 20 ml of each medium. a digital pH-meter (CONSORT nv, Parklaan 36, B2300 Turnhout, Belgium) was employed to determine the pH of the fresh immersion solutions. Every day, the solutions were freshened and kept in sealed vials to reduce evaporation and avoid microbial growth; furthermore, they were agitated to lessen particle precipitation twice a day. A 28-day staining protocol was used in this study to mimic about two years of clinical consumption of the pigmenting substances because 24 h in vitro is equal to around one month in vivo, which is adequate for long-term staining susceptibility assessment [14].

#### Color measurements

Using a white Teflon holder, specimens were positioned in the middle of a spectrophotometer’s measuring head. Each specimen from the same region received repeated measurements owing to this connection. Additionally, the system was shielded from any external light source by this configuration. Prior to exposure, each specimen’s color values (L*, a*, b*) were determined (baseline measurements) using a reflective spectrophotometer (X-Rite, model RM200QC, Neu-Isenburg, Germany). The specimens were precisely aligned with the apparatus, and the size of the aperture was fixed at 4 mm. The spectrophotometer was configured with D65 standard illumination, and the measurements were taken against a white background. Following 28 days of immersion, the same spectrophotometer was employed to record color measurements, which were carried out in the same way and under the same circumstances as the baseline readings. Three readings were recorded per specimen, and the average values were determined. The color differences of the specimens were calculated using the CIEDE2000 (∆E_00_) formula [36–38] as follows:

$$\Delta E_{00}=\sqrt{\left(\frac{\Delta L^\prime}{K_L S_L}\right)^2+\left(\frac{\Delta C^\prime}{K_C S_C}\right)^2+\left(\frac{\Delta H^\prime}{K_H S_H}\right)^2+R_{T}\left(\frac{\Delta C^\prime}{K_C S_C}\right)\left(\frac{\Delta H^\prime}{K_H S_H}\right)}$$

**Fig. 2:**
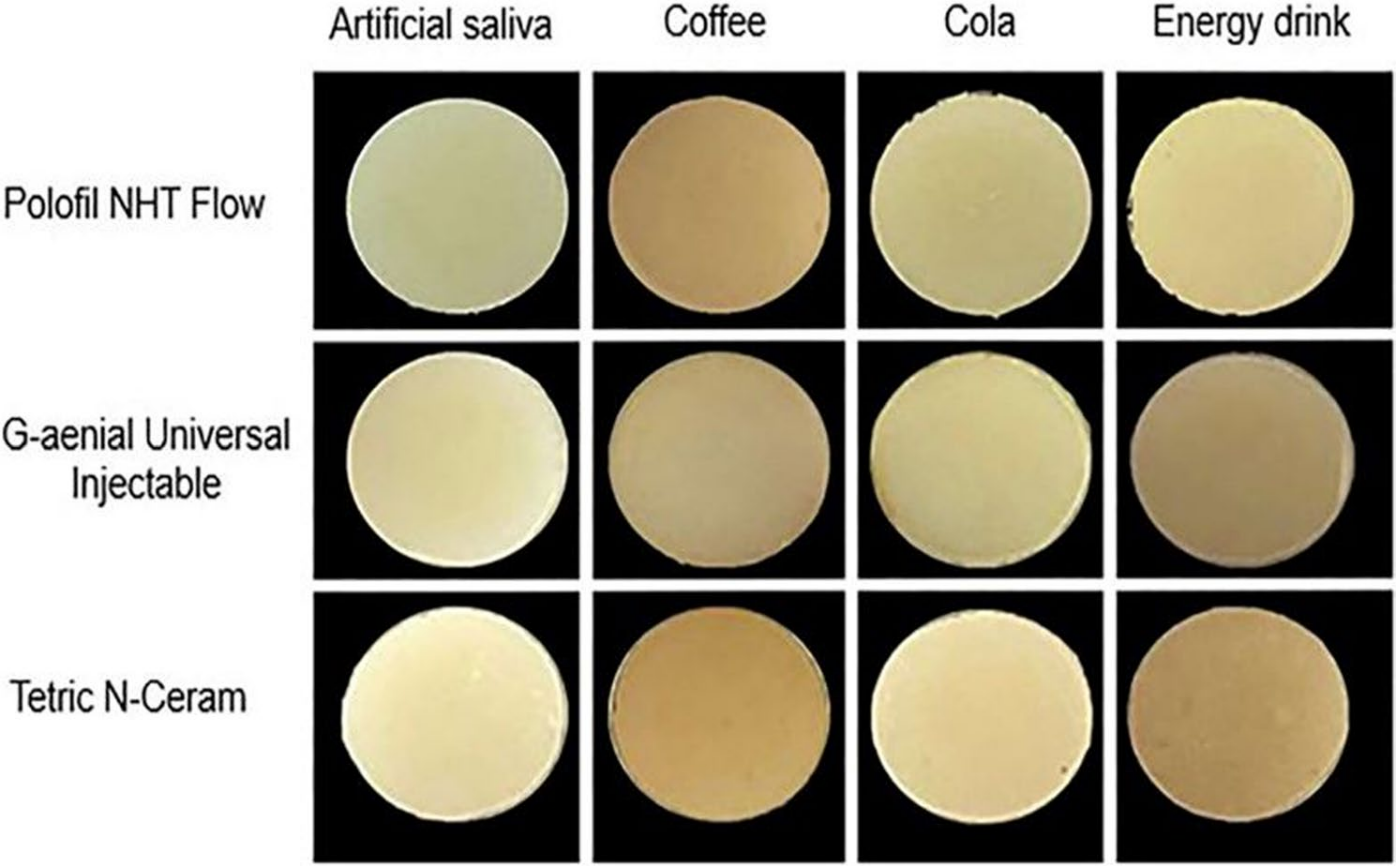
Representative specimen from each tested resin composite material showing color changes after immersion in different media

Where ∆L: differences in lightness, ∆C: differences in chroma, ∆H: differences in hue, and S_L_, S_C_, S_H_, K_L_, K_C_, and K_H_: constant coefficients. R_T_ is a rotation term that compensates for hue–chroma differences in the blue region [39]. Measurements were taken across wavelengths from 380 nm to 780 nm at 1 nm intervals. ∆E_00_ was computed using an Excel-based CIEDE2000 formula. The resulting ∆E_00_ values were compared against the perceptibility threshold, indicating the smallest color difference visible to the human eye (∆E_00_ = 0.8), and the acceptability threshold, which marks the point where color differences become visually unacceptable (∆E_00_ = 1.8) [40, 41]. The color changes of all tested resin composite materials after immersion in all immersion media are shown in Fig. 2.

### Surface roughness evaluation

**Fig. 3:**
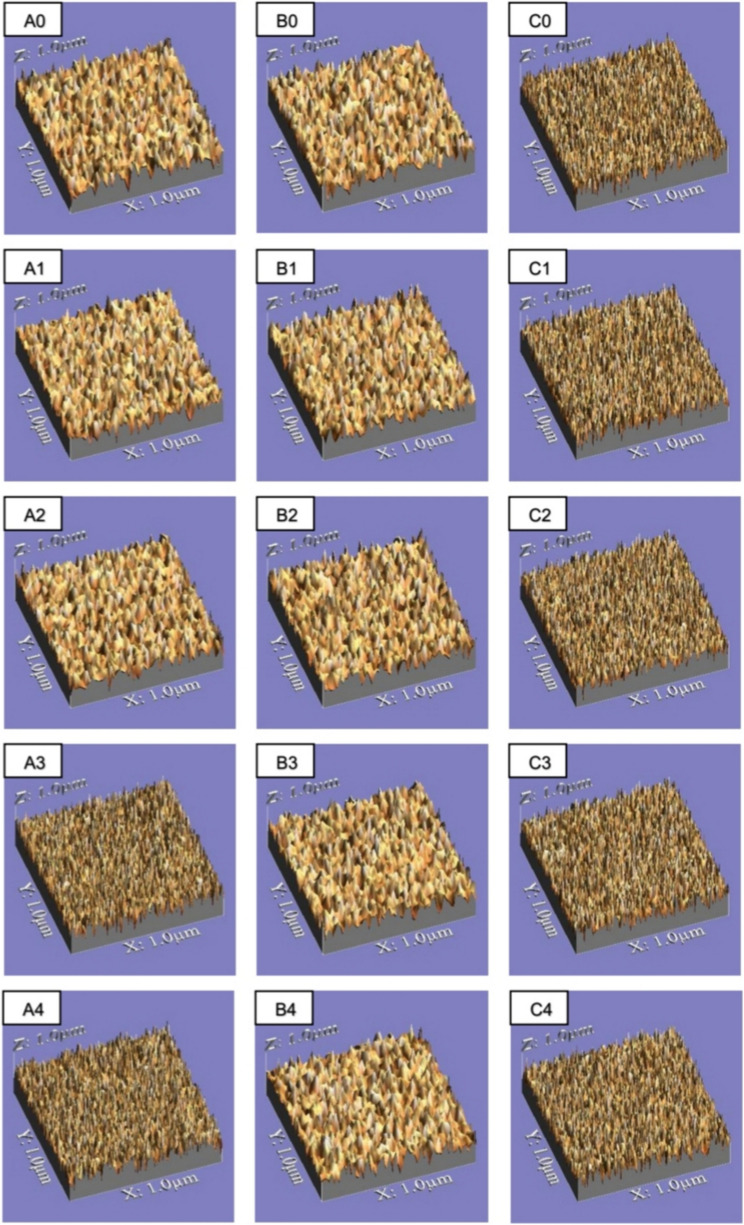
Surface roughness of representative specimens from all tested subgroups, **A** Polofil NHT Flow, **B** G-aenial Universal injectable, **C** Tetric N-Ceram, **0**: control (without immersion), **1**: artificial saliva, **2**: coffee, **3**: cola, and **4**: energy drink

Surface topography was quantitatively analyzed using an optical non-contact technique. At a magnification of 120X, with a 0.8 mm cut off and 2.4 mm evaluation length. A USB digital microscope, featuring a built-in camera (Scope Capture Digital Microscope, Guangdong, China) and linked to an IBM-compatible computer, was used for specimen examination. To ensure that the measuring area was standardized, the photos were first taken at a resolution of 1280 × 1024 pixels each, and then edited by cropping in Microsoft Office Picture Manager to 350 × 400 pixels. After analysis using WSxM software, a 3D picture of the specimens’ surface was obtained. For every sample, three 3D pictures were captured. To analyze average surface roughness (Ra) in µm, WSxM software was used [42, 43]. Surface roughness of specimens from all tested subgroups is shown in Fig. 3.

### Surface microhardness evaluation

A Vickers diamond indenter and a 20X objective lens were used; the Digital Display Vickers Micro-hardness Tester (Model HVS-50, Laizhou Huayin Testing Instrument Co., Ltd., China) was utilized to measure the specimens’ surface microhardness. For 15 s, a 50 g force was settled on the specimens’ surface [21]. For each specimen, three indentations were created on the surface, evenly distributed along a circular path with a minimum spacing of 0.5 mm. The diagonal lengths of these indentations were measured using the built-in scaled microscope, and the Vickers readings obtained were subsequently converted into microhardness values. The microhardness was calculated according to the following equation:

HV = 1.854 P/d^2^ where HV is Vickers hardness in Kgf/mm^2^, P is the load in Kgf, and d is the length of the diagonals in mm.

### Statistical analysis

All data were collected, tabulated, and analyzed using a statistical package for social studies (SPSS 25) (SPSS Inc., Chicago, Il, USA). Shapiro-Wilk test and Levene’s test were used to determine the normality of variables and homogeneity of variances for color stability, surface roughness, and surface microhardness. As the data exhibited a normal distribution, two-way ANOVA followed by Tukey’s post-hoc test was employed to compare color stability, surface roughness, and surface microhardness among the groups. Pearson correlation analysis was applied to assess the relationship between color stability and surface roughness for each tested resin composite. A significance level of *p* < 0.05 was adopted for all statistical tests.

## Results

### Color stability

Two-way ANOVA test indicated that there was a significant difference between the tested resin composites used (*p* < 0.001), and there was a significant difference between the different immersion media used (*p* < 0.001). It also revealed that there was a significant interaction between resin composites and immersion media (*p* < 0.001). Two-way ANOVA test results are shown in Table 3. The mean values and standard deviations (SDs) of ∆E_00_ values for all tested subgroups with Tukey’s post-hoc test results are shown in Table 4.

**Table 3:** Two-way ANOVA test for color stability test results

Source	Type III Sum of Squares	df	Mean Square	F	Sig.
Corrected Model	391.766	11	35.615	77.829	< 0.001
Intercept	1923.578	1	1923.578	4203.545	< 0.001
Type of resin composites	29.891	2	14.945	32.660	< 0.001
Type of immersion media	228.098	3	76.033	166.153	< 0.001
Type of resin composites* Type of immersion media	133.777	6	22.296	48.723	< 0.001
Error	49.422	108	0.458		
Total	2364.765	120			
Corrected Total	441.188	119			

**Table 4:** Comparison of mean color difference (∆E_00_ ± SD) among all tested subgroups

∆E_00_	Polofil NHT Flow	G-aenial Universal Injectable	Tetric *N*-Ceram
Artificial saliva	2.59 ± 0.35 ^Aa^	2.29 ± 0.81^Aa^	2.89 ± 0.47 ^Aa^
Coffee	4.26 ± 0.53 ^Ab^	4.50 ± 0.86 ^Ab^	4.60 ± 0.92 ^Ac^
Cola	2.63 ± 0.29 ^Aa^	2.77 ± 0.58 ^Aa^	3.36 ± 0.42 ^Aab^
Energy drink	4.88 ± 0.80 ^Ab^	9.27 ± 1.07 ^Bc^	4.02 ± 0.47 ^Abc^

Tukey’s post-hoc test results are presented as follows:

^A, B^ Different superscripted capital letters in same row indicate statistically significant differences among the tested resin composites within each tested immersion medium

^a−c^ Different superscripted lowercase letters in same column indicate statistically significant differences among the tested immersion media within each tested resin composite

There was no significant difference between all evaluated resin composites regarding artificial saliva (*p* = 0.78), coffee (*p* = 0.93), and cola (*p* = 0.09). Regarding energy drink, there was no significant difference between Polofil NHT Flow (4.88 ± 0.80) and Tetric N-Ceram (4.02 ± 0.47) (*p* = 0.17), while there were significant differences between them and G-aenial Universal Injectable (9.27 ± 1.07) (*p* < 0.05).

**Fig. 4:**
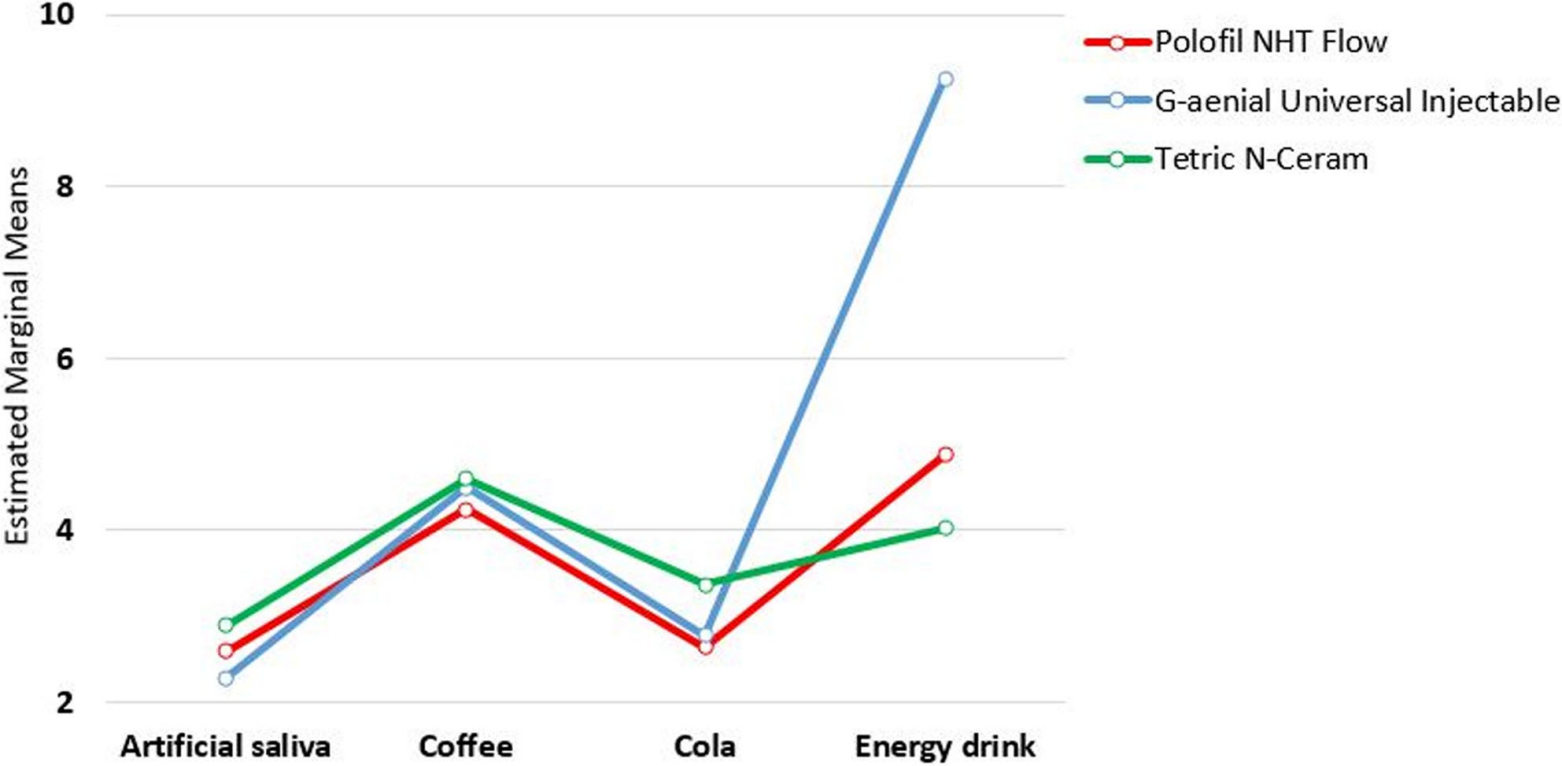
A linear chart of ∆E_00_ mean values of all tested resin composites with different immersion media

Among all examined subgroups, energy drink had the highest ∆E_00_ values, followed by coffee, cola, and artificial saliva respectively. The ∆E_00_ mean values of all tested resin composites with different immersion media are shown in Fig. 4.

### Surface roughness

Two-way ANOVA test results revealed that none of the study variables had any significant effect on surface roughness (*p* = 0.612 for the resin composites) and (*p* = 0.063 for the immersion media), and there was no significant interaction between resin composites and immersion media (*p* = 0.093). Two-way ANOVA test results are shown in Table 5. The mean values and standard deviations (SDs) of surface roughness for all tested subgroups are shown in Table 6.

**Table 5:** Two-way ANOVA test for surface roughness test results

Source	Type III Sum of Squares	df	Mean Square	F	Sig.
Corrected Model	0.095	14	0.007	3.425	< 0.001
Intercept	10.109	1	10.109	5129.623	< 0.001
Type of resin composites	0.005	2	0.003	1.344	0.612
Type of immersion media	0.052	4	0.013	6.595	0.063
Type of resin composites* Type of immersion media	0.037	8	0.005	2.361	0.093
Error	0.266	135	0.002		
Total	10.469	150			
Corrected Total	0.361	149			

**Table 6:** Comparison of mean surface roughness (Ra ± SD) in micrometer (µm) among all tested subgroups

Surface roughness (µm)	Polofil NHT Flow	G-aenial Universal Injectable	Tetric *N*-Ceram
Control (without immersion)	0.22 ± 0.08	0.22 ± 0.06	0.27 ± 0.08
Artificial saliva	0.27 ± 0.08	0.28 ± 0.05	0.27 ± 0.06
Coffee	0.27 ± 0.05	0.26 ± 0.05	0.26 ± 0.05
Cola	0.24 ± 0.07	0.23 ± 0.08	0.27 ± 0.06
Energy drink	0.32 ± 0.06	0.27 ± 0.07	0.27 ± 0.04

**Fig. 5:**
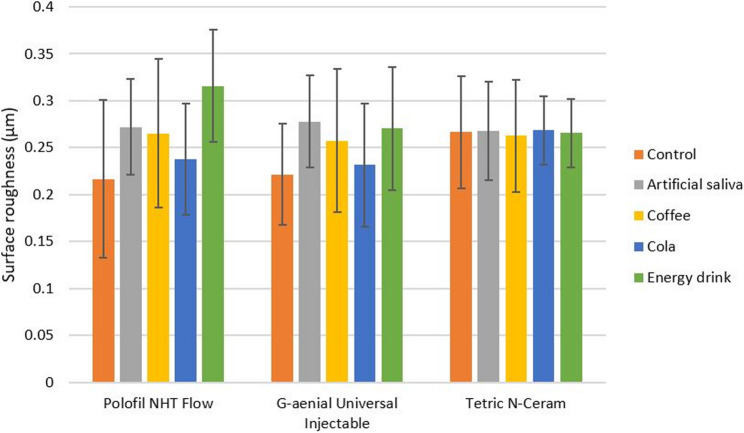
A bar chart represents mean values ± SDs of surface roughness (µm) for all tested resin composite materials with different immersion media

There was no statistically significant difference among the tested resin composites within each tested immersion medium and among control subgroups (without immersion). There was no statistically significant difference among the tested immersion media and control subgroup (without immersion) within each tested resin composite. The highest surface roughness mean value was recorded with Polofil NHT Flow, which showed an increase in surface roughness after immersion in energy drink (0.32 ± 0.06 μm) compared to the control subgroup (without immersion) (0.22 ± 0.08 μm). The mean values ± SDs of all tested resin composites with different immersion media are shown in Fig. 5.

### Surface microhardness

Two-way ANOVA test results indicated that there was a significant difference between the resin composites used (*p* < 0.001) and between the immersion media used (*p* = 0.007). However, there was no significant interaction between resin composites and immersion media (*p* = 0.130). Two-way ANOVA test results are shown in Table 7. The mean values and standard deviations (SDs) of surface microhardness for all tested subgroups with Tukey’s post-hoc test results are shown in Table 8.

**Table 7:** Two-way ANOVA test for surface microhardness test results

Source	Type III Sum of Squares	df	Mean Square	F	Sig.
Corrected Model	519.501	14	37.107	55.761	< 0.001
Intercept	720800.451	1	720800.451	1083146.489	< 0.001
Type of resin composites	35.701	2	17.850	26.824	< 0.001
Type of immersion media	451.211	4	112.803	169.509	0.007
Type of resin composites* Type of immersion media	32.590	8	4.074	6.122	0.130
Error	89.838	135	0.665		
Total	721409.791	150			
Corrected Total	609.340	149			

**Table 8:** Comparison of mean surface microhardness (VMH ± SD) in Vickers hardness number (HV) among all tested subgroups

Surface microhardness (HV)	Polofil NHT Flow	G-aenial Universal Injectable	Tetric *N*-Ceram
Control (without immersion)	73.56 ± 0.79 ^Ba^	70.74 ± 0.85 ^Aa^	73.42 ± 0.96 ^Ba^
Artificial saliva	69.04 ± 1.49 ^ABb^	67.93 ± 1.27 ^Ab^	69.37 ± 1.15 ^Bb^
Coffee	68.60 ± 0.90 ^Ab^	68.90 ± 1.20 ^Ab^	68.90 ± 0.90 ^Ab^
Cola	67.93 ± 1.76 ^Ab^	67.75 ± 0.80 ^Ab^	68.45 ± 1.23 ^Ab^
Energy drink	68.50 ± 1.00 ^Ab^	67.40 ± 1.10 ^Bb^	68.10 ± 1.10 ^Ab^

Tukey’s post-hoc test results are presented as follows:

^A, B^ Different superscripted capital letters in same row indicate statistically significant differences among the tested resin composites within each tested immersion medium and among control subgroups (without immersion)

^a, b^ Different superscripted lowercase letters in same column indicate statistically significant differences among the tested immersion media and control subgroup (without immersion) within each tested resin composite

Regarding control subgroups, there was no statistically significant difference between Polofil NHT Flow (73.56 ± 0.79 HV) and Tetric N-Ceram (73.42 ± 0.96 HV) (*p* = 0.31), while there were statistically significant differences between them and G-aenial Universal Injectable (70.74 ± 0.85 HV) (*p* < 0.05). Polofil NHT Flow, G-aenial Universal Injectable, and Tetric N-Ceram showed significant decreases in microhardness after immersion in all immersion media in comparison to the control subgroups (*p* < 0.05).

**Fig. 6:**
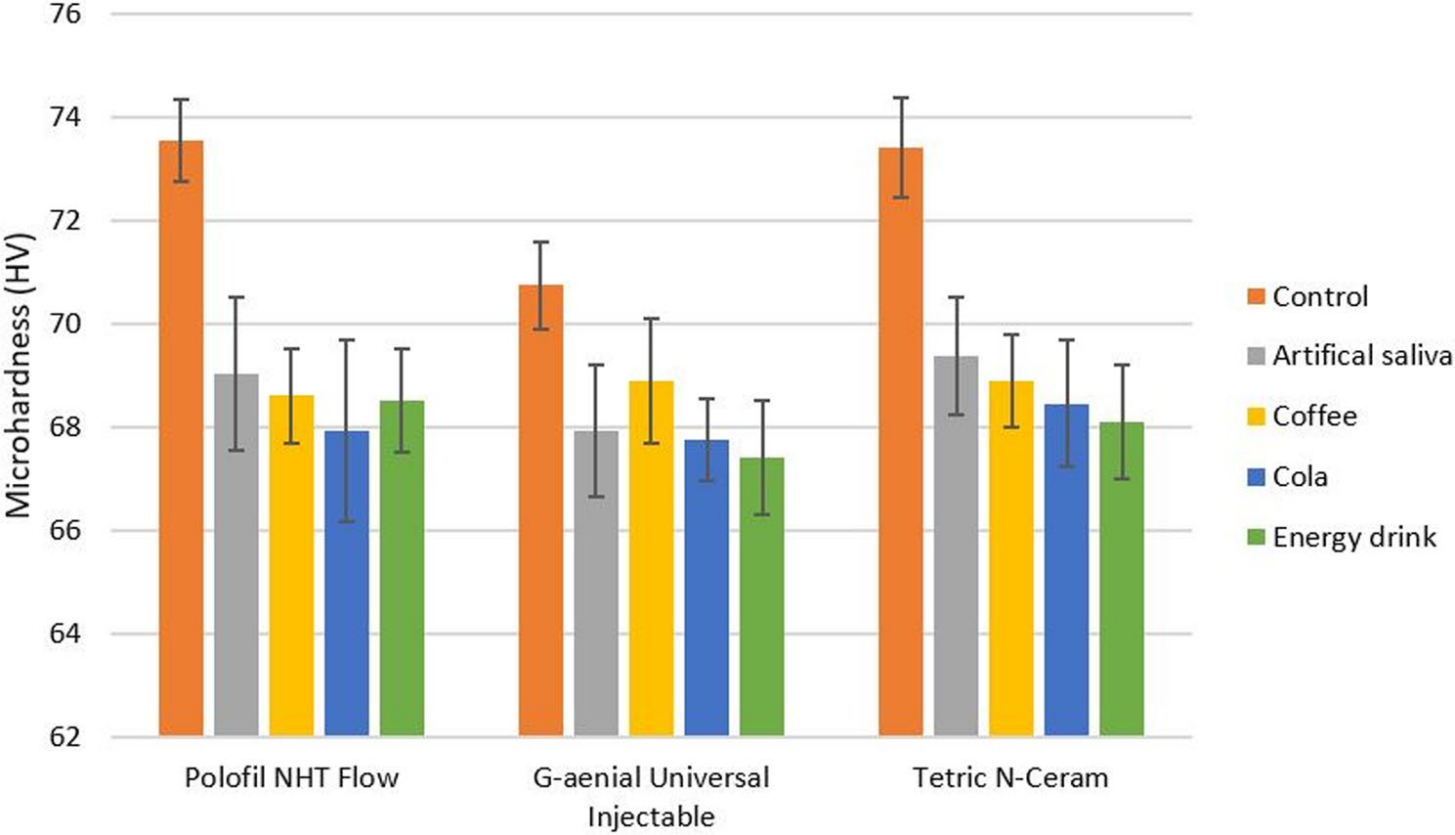
A bar chart represents mean values ± SDs of surface microhardness (HV) for all tested resin composite materials with different immersion media

After immersion in artificial saliva, there was a significant difference in microhardness between G-aenial Universal Injectable (67.93 ± 1.27 HV) and Tetric N-Ceram (69.37 ± 1.15 HV) (*p* = 0.01), while there were no statistically significant differences between them and Polofil NHT Flow (69.04 ± 1.49 HV) (*p* > 0.05). There was no significant difference among all tested resin composite materials after immersion in coffee (*p* = 0.60), and after immersion in cola (*p* = 0.34). After immersion in energy drink, there was no significant difference between Polofil NHT Flow (68.50 ± 1.00 HV) and Tetric N-Ceram (68.10 ± 1.10 HV) (*p* = 0.08), while there were significant differences between them and G-aenial Universal Injectable (67.40 ± 1.10 HV) (*p* < 0.05). The mean values ± SDs of all tested resin composite materials with different immersion media are shown in Fig. 6.

### Correlation between color stability and surface roughness

**Fig. 7:**
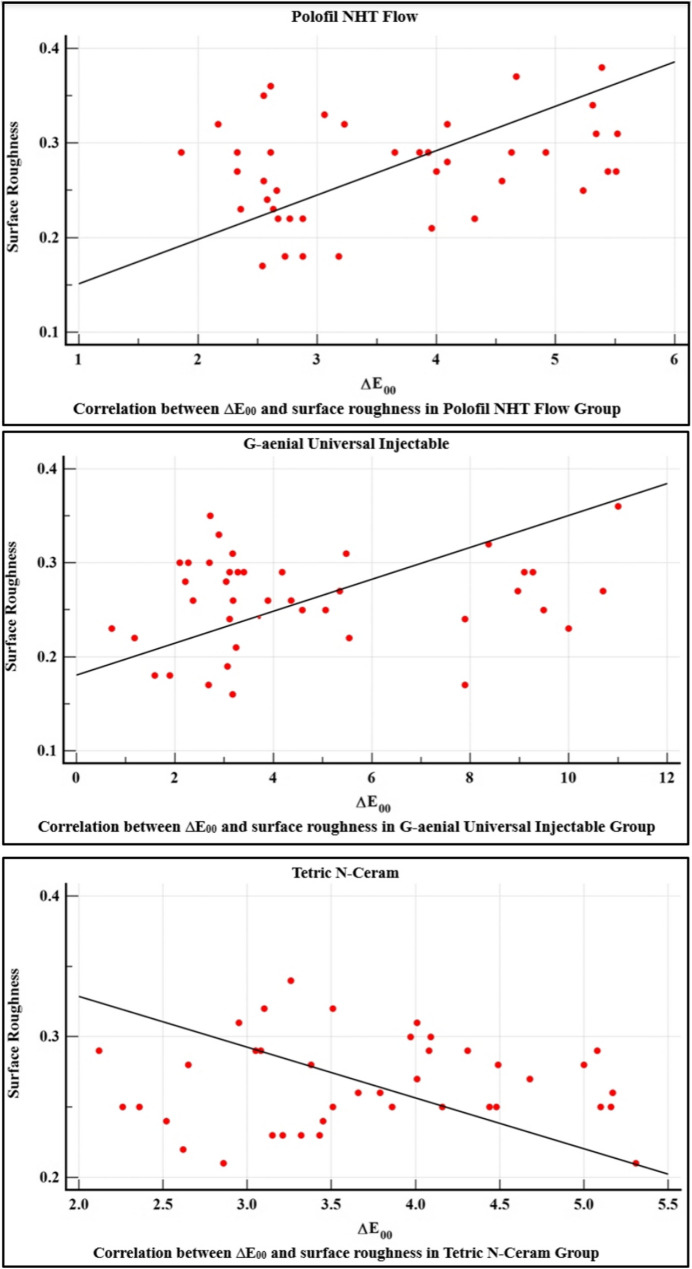
Correlation between ∆E_00_ and surface roughness for each tested resin composite

Within the Polofil NHT Flow group, surface roughness and color stability were positively correlated. However, there was no correlation between color stability and surface roughness within G-aenial Universal Injectable and Tetric N-Ceram groups, as shown in Table 9. The correlations between ∆E_00_ and surface roughness for each tested resin composite are shown in Fig. 7.

**Table 9:** Correlation between color stability and surface roughness within each tested resin composite material

Correlation between color stability and surface roughness		∆E_00_	
		**R**	***p*** **-value**
Polofil NHT Flow	**Surface roughness**	0.31	0.0497*
G-aenial Universal Injectable		0.19	0.239
Tetric N-Ceram		−0.01	0.973

R: Pearson’s correlation coefficient, *: significance < 0.05

## Discussion

In the current study, the type of the tested resin composite affected the color stability and surface microhardness, while it had no impact on surface roughness; thus, the first null hypothesis was partially rejected. The type of immersion medium affected the color stability and surface microhardness, while it had no impact on surface roughness; thus, the second null hypothesis was partially rejected.

Flowable resin composites were launched into the market with great expectations to address the drawbacks of packable resin composites, such as material adaptability. These materials can flow thanks to the low filler loading and high monomer content of these flowable resin composites, although frequently at the expense of inferior physical qualities [44]. It is generally acknowledged that a resin composite’s mechanical behavior improves with increasing filler content, which in turn increases the restoration’s potential longevity. As a result, flowable resin composites with a very high filler content, combining both flowability and better mechanical properties, have arisen, and manufacturers may emphasize the filler content as a marketing point [45].

By incorporating nanoparticles into resin composites, it may be possible to create flowable materials having better flow characteristics and higher mechanical properties, esthetics, and polishability than those seen in earlier flowable resin composites. According to nanoscience, the materials’ structure can be manipulated at the nanoscale, where fillers are 40 nm–0.4 μm in size. This enabled the improvement of the mechanical properties and filler load without compromising its viscosity [3, 5]. These properties make them suitable for a range of indications, including small Class I and II restorations, Class V lesions, and as liners or initial layers beneath conventional resin composites.

To quantify the shade changes both before and after the specimens had been immersed in staining solutions; a spectrophotometer was used, which offers a more reliable alternative to visual evaluation by eliminating subjective bias. The CIEDE2000 (∆E_00_) formula was used as it is a refined formula for color difference calculation, which shows improved conformity relative to ∆Eab, enhancing the correlation between calculated color differences and those perceived visually. This is because ∆E_00_ accounts for not only differences in lightness, chroma, and hue through specific weighting functions, but also incorporating an interaction term between chroma and hue [39]. In dentistry, the 50:50% acceptability threshold is frequently employed to evaluate estimated color differences in the CIE L, a*, b* system with visual assessments. This threshold corresponds to the level at which 50% of observers perceive two colors as acceptably matching. In this study, the acceptability threshold was applied to analyze the results and assess their clinical relevance [4].

A noncontact digital profilometer microscope was employed for surface roughness measurements since it can utilize a laser to scan the surface and produce a 3-D surface map without causing any damage to the specimens. This makes it a quick and simple evaluation approach [46]. In the present study, the Vickers microhardness test was utilized for the assessment of surface microhardness of resin composites used, because it is considered one of the most popular tests in this aspect [22]. Its benefits include being easy to use, reproducibility, and minimal damage to the study samples. This is because the Vickers test employs a diamond indenter with a square-based pyramidal shape, proving to be appropriate for a broad variety of materials, including resin composites. Additionally, the Vickers test provides precise measurements, which are crucial for evaluating the mechanical characteristics of resin composite.

According to the study results, G-aenial Universal Injectable showed the highest significant change in ∆E_00_ values among all groups, regardless of the solution type, compared to Polofil NHT Flow and to Tetric N-Ceram. This result may be due to the lower amount of inorganic fillers (69% wt.) of G-aenial Universal Injectable compared to Polofil NHT Flow (76% wt.) and Tetric N-Ceram (81% wt.). Urethane dimethacrylate (UDMA) was considered to have a decreased rate of water absorption and to be a pigment-resistant co-monomer, hence boosting the stability of color [12, 47, 48]. As stated by Sideridou et al. [1] the maximum water sorption capacity is attributed to triethylene glycol dimethacrylate (TEGDMA), followed by diglycidyl ether dimethacrylate (BisGMA), and UDMA. Since Tetric N-Ceram is the only resin composite that does not have TEGDMA in its organic matrix in addition to having UDMA besides to its high filler content (81% wt.), this may explain why Tetric N-Ceram had the least color change.

The present study also reflected the influence of the composition of immersion media on discoloration of resin composite, where energy drink showed a significant highest discoloration among all subgroups, followed by coffee, cola, and artificial saliva. The strong discoloration impact of the energy drink (Red Bull) might have been caused by the use of artificial coloring additives. The energy drink has two artificial dye pigments: INS 110 (Sunset Yellow FCF), which is a yellow-orange color dye, and INS 102 (Tartrazine), which is a lemon yellow color dye. Another yellow-orange component that contributes to the yellow color of vitamin supplements is vitamin B2 (riboflavin). The artificial pigments and the beverages’ low pH could be two significant external causes for the color change.

The results of this study agree with Tanthanuch et al. [28] who examined the color alterations of tooth-colored restorative materials during exposure to energy drinks and sports drinks. They found that the energy drink displayed a significant increase in color change and surface roughness and a decrease in surface hardness when compared to a sports drink. This is consistent with Azer et al. [9] who concluded that although the coloring agents in the consumed foods and drinks are attributed to external factors, the degree of the color change is also influenced by the low pH of the foods and drinks, rather than the dietary pigment alone.

Immersion in artificial saliva caused the least amount of discoloration in all tested resin composites, which is consistent with other investigations [6, 11, 27]. Given that artificial saliva lacks pigments, the color changes could be the result of the matrix’s water sorption, which causes the polymer to swell and plasticize, as well as the formation of interfacial gaps between the resin matrix and fillers that permit discoloration in addition to stain penetration [6].

In the current research, coffee had a higher discoloring impact than cola. These findings are consistent with previous studies that have demonstrated that compared to cola, coffee has more chromogens and yellow pigments with a smaller molecular weight, which causes considerable staining on resin composites [13, 14, 26]. In the present study, all tested groups were kept in an incubator at 37 ± 1 °C. This might explain why coffee had a lesser coloring effect than energy drink, as coffee produces higher discoloration and causes higher degradation to the polymeric matrix at higher temperatures. This agrees with Silva et al. [31] who concluded that hot temperatures (65–80 °C) in tea and coffee result in color deterioration and water sorption, leading to the degradation of the surface of the polymeric matrix. Additionally, coffee showed a stronger effect on the discoloration of the resin composite than cola and water according to Ertaş et al. [14], which is in line with the results of the current study.

Studies have indicated that particle dimension and structure have a greater influence on roughness than particle hardness. The wear rates of resin composite restorations are reduced and the smoothness is enhanced when the filler content is increased while the size of the filler particle is reduced. Additionally, the onset of restorative material surface degradation is significantly influenced by the pH of the oral environment and the duration of exposure to the consumed beverages [7, 23, 29]. All resin composites used in the current study are nanohybrid with small filler particle size; this may explain why there was no significant difference in surface roughness among the subgroups. The standardization of the preparation process for specimens could be another cause [10].

In the present study, Polofil NHT Flow revealed the highest surface roughness following its immersion in energy drink; this could be due to the acidity of Red Bull, which contains citric acid, which affects the resin matrix in Polofil NHT Flow, as acidic drinks with low pH values and citric acid have a high erosive potential [30]. Polofil NHT Flow’s resin matrix contains Bis-GMA, TEGDMA, and UDMA. Bis-GMA absorbs less water than TEGDMA, but absorbs more water than the resins made by UDMA and Bis-EMA. Because of the comparatively soft resin matrix, filler particles protrude from the surface after being exposed to the highly acidic beverage (Red Bull), which causes them to leach out preferentially [14]. This also explains why Tetric N-Ceram showed the least surface roughness after immersion in energy drink.

This study agrees with Tanthanuch et al. [28] who carried out an investigation about the effectiveness of energy drinks and sports drinks on the surface properties of different restorative materials. They discovered that the surface roughness substantially increased, as the drinks with the lowest pH value and the most erosive properties resulted in the greatest roughness value during the immersion period. UDMA exhibits poor solubility and water absorption properties [49]. In contrast, TEGDMA is a water-absorbing hydrophilic monomer. Due to an increase in the hydrophilicity of the water absorption, the storage modulus of resin composites containing TEGDMA reduces as the immersion time increases. It is believed that hydrophilic groups, like the ethoxy group in TEGDMA, exhibit affinity for water molecules via forming hydrogen bonds with oxygen [32]. Furthermore, the restorative materials that contain Bis-GMA or TEGDMA increase the water uptake from 0 to 1.0% by weight, depending on the percentage of the added Bis-GMA or TEGDMA [14]. This is in line with the findings of De Moraes et al. [19].

Regarding control subgroups in the current study, G-aenial Universal Injectable showed a lower microhardness mean value (70.74 ± 0.85 HV) than Polofil NHT flow (73.56 ± 0.79 HV) and Tetric N-Ceram (73.42 ± 0.96 HV). Researchers have long known that as the content of filler increases, higher microhardness values are anticipated, which could explain this result [18, 20, 24, 25]. According to previous studies, restorative materials degrade in acidic situations, which lowers their hardness [50, 51]. It has been observed that a solution’s erosive potential depends not only on its low pH but also on kind of acid, immersion period in acidic liquids, and the composition of beverage. The degradation with citric acid-containing solutions (as Red Bull) is dependent on the chelation and diffusion between the eluted particles and the acid anions [52]. This explains the results in the current study, where immersion in cola (containing phosphoric acid) caused a substantial decrease in surface microhardness for all tested resin composites. Energy drink containing citric acid also caused a significant decrease in surface microhardness. These results are in agreement with Khan et al. [53].

The current study results align with Abu-Bakr et al. [54] who reported that the surface of resin composites was significantly damaged by various beverages with varying pH values, and this surface deterioration was more noticeable at lower pH values. They clarified that chemical degradation resulting from filler-matrix debonding, hydrolytic degradation of the bond between the silane and the filler particles, or hydrolytic deterioration of the fillers can cause the resin composite’s mechanical and physical characteristics to deteriorate [55]. The microstructure of the resin composite was altered by gradual deterioration, creating pores. When water penetrates polymer chains through porosity and intermolecular space, it can cause the degradation of polymer chain bonds [55]. This subsequently results in the erosion of filler particles and the dissolving of monomer, which degrades the physical qualities of resin composites.

The separation of the polymer chains by molecules that do not form a primary chemical bond chains leads to a decrease in resin composites’ physical qualities [56]. Water aggregation at the filler-matrix interface either speeds up the disintegration of inorganic particles or causes the pre-existing superficial defects to gradually become more noticeable. This negative impact on the resin composite’s polymeric network may alter its structure chemically and physically. Saliva’s moisture and water cause peeling stress, a plasticizing influence on the structure, and the debonding of filler from the matrix [57]. Silva et al. [58] concluded that after 30 days of immersion in artificial saliva, the resin composite’s microhardness decreased, and this is consistent with current research.

In the present study, there was a positive correlation within the Polofil NHT group between surface roughness and color change, as an increase in surface roughness was associated with a corresponding increase in color change, indicating a reduction in the color stability of the resin composite. This agrees with Chowdhury et al. [59] who evaluated the surface roughness and color stability of nanohybrid resin composites after periodic exposure to tea, coffee, and cola.

This in vitro study offers several strengths. The controlled laboratory environment allows for precise standardization of variables such as immersion time, temperature, and specimen size. Furthermore, the use of highly filled flowable nanohybrid resin composites, modern restorative materials with widespread clinical application, increases the relevance of the results to dental practice. Additionally, the use of multiple common media such as artificial saliva, coffee, cola, and energy drink provides a realistic simulation of dietary exposures. The evaluation of three important material properties, color stability, surface roughness, and surface microhardness, allows for a comprehensive assessment of the material’s behavior under erosive and staining conditions.

This study also had some limitations; the in vitro investigation does not fully replicate the dynamic and complex nature of the oral environment, which includes salivary enzymes, bacterial biofilm, temperature fluctuations, and mechanical forces such as brushing and mastication. The immersion protocol may oversimplify real-life exposure, as continuous immersion does not reflect intermittent contact with beverages. The use of artificial saliva, lacks the biological complexity of natural human saliva. The relatively short duration of immersion may also limit the extrapolation of results to long-term clinical performance.

## Conclusions

Within the limitations of this study the following conclusions can be drawn:

1. For color stability, energy drink and coffee had the most negative effect on all tested resin composites.
2. Immersion in energy drink caused the highest discoloration with G-aenial Universal Injectable and the highest surface roughness with Polofil NHT Flow.
3. All tested resin composites showed a decrease in surface microhardness following immersion in all tested immersion media.
4. Dentists should notify patients with resin composite restorations about the discoloration, increased plaque accumulation due to the rough surface, and decrease in surface microhardness of the restoration that may occur after extended periods of consumption of different beverages.

## Data Availability

The data that support the findings of this study are available from the corresponding author upon reasonable request.

## Abbreviations

RBCs: Resin-based composites
NHT: Nanohybrid technology
ANOVA: Analysis of variances
∆E_00_: Color change
Bis GMA: Bisphenol A-diglycidyl dimethacrylate
TEGDMA: Triethylene glycol dimethacrylate
HEDMA: 2-Hydroxyethyl dimethacrylate
UDMA: Urethane di-methacrylate
Bis-MEPP: Bisphenol A ethoxylate dimethacrylate
Bis-EMA: Bisphenol A ethoxylated dimethacrylate
LED: Light emitting diode
L: The chromaticity coordinate in the white-black axis
a: The chromaticity coordinate in the red-green axis
b: The chromaticity coordinate in the yellow-blue axis
CIE: Commission Internationale de l’Eclairage
3D: 3 Dimensions
µm: Micrometer
Ra: Arithmetic roughness mean
VMH: Vickers microhardness
HV: Vickers hardness number
SDs: Standard deviations
